# Do GPCRs constitute the target of 30% of newly approved drugs in Germany?

**DOI:** 10.1007/s00210-025-04548-3

**Published:** 2025-11-06

**Authors:** Sophia Schröer, Roland Seifert

**Affiliations:** Hannover Medical School, Institute of Pharmacology, Carl-Neuberg-Str. 1, 30625 Hannover, Germany

**Keywords:** GPCR, G-protein-coupled receptor, Drug target, 30% GPCR, WHO Model List of Essential Medicines, Rote Liste, Newly approved drugs in Germany

## Abstract

G protein-coupled receptors (GPCRs) are often stated to constitute the target of 30% (or even more) of all approved drugs. This number, probably first mentioned in (https://pubmed.ncbi.nlm.nih.gov/12209152/) and later has been often cited by many other studies and in some studies even taken for granted, but is this true? In this study, the number of approved drugs acting on GPCRs was analyzed in relation to the total number of approved drugs in Germany. Newly approved drugs in the years 1987–2023 were analyzed. Of the newly approved drugs in Germany in this period, 209 out of 1110 drugs (19%) act primarily on GPCRs. The most common drug target during this period were enzymes (mostly inhibitors), followed by receptor agonists and antagonists. Among all receptor classes, GPCRs are the most common drug target. In recent years, there has been an increase in antibody approvals, making them the fourth most common drug class in the time from 1987–2023. For the years 1999/2000, 2002 and 2023, all drugs listed in the WHO Model List of Essential Medicines were examined regarding targeting GPCRs. In none of the years do more than 15% of the drugs act on GPCRs. 63 of 293 drugs (22%) of the drug list (version 1.0) and 103 of 464 (22%) of version 2.0 of the Institut für Medizinische und Pharmazeutische Prüfungsfragen (Institute for Medical and Pharmaceutical Review Questions) in Germany were identified as GPCR drugs. All drugs of the Rote Liste (Red List) from 2002 and 2023 were examined regarding targeting GPCRs, resulting in a share of 20%. The frequently cited 30% in the drug studies conducted to date mostly refers to FDA-approved drugs. Therefore, the proportion of GPCR-mediated FDA approved drugs does not match the newly approved drugs in Germany or other analyzed sources. This study sends a note of caution to researchers when using data without further specification or precise context in the literature. Such numbers should be analyzed critically and not taken for granted in every situation.

**Supplementary Information:**

The online version contains supplementary material available at 10.1007/s00210-025-04548-3.

## Introduction

Every year, many new drugs are approved worldwide. The FDA publishes an overview of newly approved drugs each year (https://www.fda.gov/drugs/development-approval-process-drugs/novel-drug-approvals-fda, last accessed 01.08.2025). The FDA-approved drugs are reviewed on an annual basis (Kayki-Mutlu and Michel 2021)(Kayki-Mutlu et al. 2022)(Kayki-Mutlu et al. 2023) (Kayki-Mutlu et al. 2024). Papapetroupolos et al. (2024), and Topouzis et al. (2025) have recently joined the review of FDA-approved drugs and included drugs approved by the EMA and MHRA. For Germany, newly approved drugs are listed in the AVR Schwabe et al. (1998, 1999, 2000, 2001, 2002, 2003, 2004, 2006, 2007, 2008, 2010a, 2010b, 2011a, 2011b, 2012, 2013, 2014, 2015, 2016, 2017, 2018, 2019, 2020), (Ludwig et al. 2022, 2023, 2024, 2025) and in book series such as “Neue Arzneimittel” (Fricke and Klaus 1989, 1988, 1990, 1991,1992, 1994, 1995, 1997, 1999, 2000).

Common drug targets are enzymes, ion channels, transporters and receptors (https://www.guidetopharmacology.org, last accessed 02.08.2025). Receptors are classified into ligand-gated ion channels, nuclear receptors, tyrosine kinase-linked receptors and GPCRs (https://www.guidetopharmacology.org, last accessed 02.08.2025). (Bedair and Mansour, 2021), (Latham et al., 2024), (Hauser et al. 2017).

To our knowledge, there is no comprehensive analysis of the newly approved drugs in Germany over the past decades. The German drug market is one of the fastest launching countries and has a high availability of new pharmaceuticals (Büssgen and Stargardt 2022). Pharmaceutical sales reach EUR 59.8 billion in 2023 (https://www.gtai.de/en/invest/industries/healthcare-market-germany/pharmaceutical-industry, last accessed 10.06.2025). This makes Germany the largest pharmaceutical market in Europe (Nussli et al. 2024) and an important international pharmaceutical hub (https://altios.com/publication/the-pharmaceutical-industry-in-germany/, last accessed 06.06.2025).

This prompted us to analyze the drug approvals in Germany over the past four decades. We were particularly interested in GPCRs since many sources quote that GPCR drugs account for 30–35% of the newly approved drugs, numbers sometimes reaching 40–50%. 69 of such statements are listed in Table 1. Specific sources for these percentages are often not provided. Therefore, we asked the question whether these percentages for specific databases and newly approved drugs in Germany are true. Additionally, we compared the percentages of FDA approved GPCR-drugs with the German drug market. Lastly, for comparison, we also analyzed the WHO essential drug list (https://www.who.int/groups/expert-committee-on-selection-and-use-of-essential-medicines/essential-medicines-lists, last accessed 02.08.2025), the Rote Liste (https://www.rote-liste.de, last accessed 02.08.2025) and the IMPP drug list (https://www.mhh.de/fileadmin/mhh/zentrum-pharmakologie-toxikologie/download/IMPP-Arzneistoffliste.pdf, last accessed 02.08.2025) regarding the percentage of GPCR drugs.

**Table 1:** Sources providing the percentage of GPCR drugs. Sources are sorted chronologically starting with the newest source

Number	Authors	Share of GPCR drugs	Year	Source of the percentage	Cited source	Data base	Mention
1	(Wikipedia.org n.d.)	30%	2023	https://de.wikipedia.org/wiki/G-Protein-gekoppelte_Rezeptoren (last accessed 23.07.2025)	Joachim Rassow, Karin Hauser, Roland Netzker, Rainer Deutzmann: Biochemie (= Duale Reihe). 4. Auflage. Thieme, Stuttgart 2016, ISBN 978–3-13–125,354-5, S. 561	All prescripted drugs	Main
2	(Yasi et al. 2020)	30%	2023	Pubmed (ScienceDirect, https://www.sciencedirect.com/science/article/abs/pii/S095816692030077X, last accessed 23.07.2025)	cited Sriram, K., and Insel, P.A. (2018). G Protein-Coupled Receptors as Targets for Approved Drugs: How Many Targets and How Many Drugs? Mol. Pharmacol. 93, 251–258. DOI: 10.1124/mol.117.111062.	FDA	Abstract, Introduction
3	(Schmitz and Roth 2023)	30%	2023	Pubmed (American journal of physiology, https://pubmed.ncbi.nlm.nih.gov/37067459/, last accessed 24.07.2025)	cited A.S. Hauser, M.M. Attwood, M. Rask-Andersen, H.B. Schioth, D.E. Gloriam Trends in GPCR drug discovery: new agents, targets and indications Nat Rev Drug Discov, 16 (2017), pp. 829–842	Approved medications	Abstract, Highlights
4	(Majumdar et al. 2024)	30%	2023	Pubmed (ScienceDirect, https://www.sciencedirect.com/science/article/abs/pii/S1359644623003641?via%3Dihub, last accessed 24.07.2025)	cited A.S. Hauser, M.M. Attwood, M. Rask-Andersen, H.B. Schioth, D.E. Gloriam Trends in GPCR drug discovery: new agents, targets and indications Nat Rev Drug Discov, 16 (2017), pp. 829–842	Approved drugs	Abstract
5	Leipzig University Hospital (Scholz et al. 2023)	40%	2023	Website (University Leipzig/Nature, https://www.nature.com/articles/s41586-023-05802-5.epdf?sharing_token=bF0CiNtN7zQHG7RodgO5LNRgN0jAjWel9jnR3ZoTv0MLHsY9vwhU03TPhy-mrVjYhECQaq0Ho8YUIUvO_WQltZlV5S-Z4YWZa5vSGXsffDk0nOEqFiNhVZuQKA4Qi8K4_mg0mjWAvUJh4vsycKJZVydYzD9whIv0yYqIX6efI48%3D,last accessed 24.07.2025)	NA	All drugs	Press release
6	(Di Marino et al. 2023)	34%	2023	Nature (https://www.nature.com/articles/s41467-023-42082-z, last accessed 24.07.2025)	cited Sriram, K., and Insel, P.A. (2018). G Protein-Coupled Receptors as Targets for Approved Drugs: How Many Targets and How Many Drugs? Mol. Pharmacol. 93, 251–258. DOI: 10.1124/mol.117.111062.	Marketed drugs	Introduction
7	(Amer et al. 2023)	30%	2023	Nature (https://www.nature.com/articles/s41598-023-36855-1, last accessed 24.07.2025)	1. cited Congreve, M., de Graaf, C., Swain, N. A. & Tate, C. G. Impact of GPCR structures on drug discovery. Cell 181, 81–91. https://doi.org/10.1016/j.cell.2020.03.003 (2020) 2. cited A.S. Hauser, M.M. Attwood, M. Rask-Andersen, H.B. Schioth, D.E. Gloriam Trends in GPCR drug discovery: new agents, targets and indications Nat Rev Drug Discov, 16 (2017), pp. 829–842, 3. cited Santos, R. et al. A comprehensive map of molecular drug targets. Nat. Rev. Drug Discov. 16, 19–34. https://doi.org/10.1038/nrd.2016.230 (2017).	Approved drugs on the market	Introduction
8	(Anantakrishnan and Naganathan 2023)	More than 30%	2023	Nature (https://www.nature.com/articles/s41467-023-35790-z, last accessed 24.07.2025)	cited Katritch, V., Cherezov, V. & Stevens, R. C. Diversity and modularity of G protein-coupled receptor structures. Trends Pharm. Sci. 33, 17–27 (2012). (https://pubmed-1ncbi-1nlm-1nih-1gov-1v67xgb0y006b.mhh.hh-han.com/22032986/	Clinically approved drugs	Introduction
9	(Kobayashi, K. et al. 2023)	More than 30%	2023	Nature (https://www.nature.com/articles/s41586-023-06169-3#citeas, last accessed 24.07.2025)	cited A.S. Hauser, M.M. Attwood, M. Rask-Andersen, H.B. Schioth, D.E. Gloriam Trends in GPCR drug discovery: new agents, targets and indications Nat Rev Drug Discov, 16 (2017), pp. 829–842	Marketed drugs	Main
10	(Wong et al. 2023)	35%	2023	Nature (https://www.nature.com/articles/s41392-023-01427-2, last accessed 24.07.2025)	cited A.S. Hauser, M.M. Attwood, M. Rask-Andersen, H.B. Schioth, D.E. Gloriam Trends in GPCR drug discovery: new agents, targets and indications Nat Rev Drug Discov, 16 (2017), pp. 829–842, cited Sriram, K., and Insel, P.A. (2018). G Protein-Coupled Receptors as Targets for Approved Drugs: How Many Targets and How Many Drugs? Mol. Pharmacol. 93, 251–258. DOI: 10.1124/mol.117.111062.	FDA listed	Introduction
11	(Oron-Herman et al. 2023)	A third	2023	Nature (https://www.nature.com/articles/s41598-023-47877-0, last accessed 24.07.2025)	cited A.S. Hauser, M.M. Attwood, M. Rask-Andersen, H.B. Schioth, D.E. Gloriam Trends in GPCR drug discovery: new agents, targets and indications Nat Rev Drug Discov, 16 (2017), pp. 829–842	FDA approved	Introduction
12	(Dahl et al. n.d.)	30%	2023	Science (https://www.science.org/doi/10.1126/sciadv.adf9297, last accessed 24.07.2025)	cited R. Santos, O. Ursu, A. Gaulton, A. P. Bento, R. S. Donadi, C. G. Bologa, A. Karlsson, B. al-Lazikani, A. Hersey, T. I. Oprea, J. P. Overington, A comprehensive map of molecular drug targets. Nat. Rev. Drug Discov. 16, 19–34 (2017). Cited R. C. Stevens, V. Cherezov, V. Katritch, R. Abagyan, P. Kuhn, H. Rosen, K. Wüthrich, The GPCR Network: A large-scale collaboration to determine human GPCR structure and function. Nat. Rev. Drug Discov. 12, 25–34 (2013)	Therapeutic drugs	Introduction
13	(Guo et al. 2022)	A quarter	2022	Pubmed (American journal of physiology, https://pubmed.ncbi.nlm.nih.gov/35816640/, last accessed 24.07.2025)	cited A.S. Hauser, M.M. Attwood, M. Rask-Andersen, H.B. Schioth, D.E. Gloriam Trends in GPCR drug discovery: new agents, targets and indications Nat Rev Drug Discov, 16 (2017), pp. 829–842	Global marketed therapeutic drugs	Abstract
14	(Ueda et al. 2022)	30%	2022	Pubmed (ScienceDirect, https://pubmed.ncbi.nlm.nih.gov/35168190/, last accessed 24.07.2025)	NA	Modern drugs	Abstract, Introduction
15	(Van Baelen et al. 2022)	30%	2022	Pubmed (Frontiers, https://pubmed.ncbi.nlm.nih.gov/35198603/, last accessed 24.07.2025)	cited Hauser, A. S., Attwood, M. M., Rask-Andersen, M., Schiöth, H. B., and Gloriam, D. E. (2017). Trends in GPCR Drug Discovery: New Agents, Targets and Indications. Nat. Rev. Drug Discov. 16 (12), 829–842. doi:10.1038/nrd.2017.178	Therapeutic targets	Abstract, Introduction, conclusion
16	(Piper et al. 2022)	30%	2022	Pubmed (ScienceDirect, https://pubmed.ncbi.nlm.nih.gov/35671790/, last accessed 24.07.2025)	NA	Approved drugs	Abstract
17	(Wess 2022)	30–35%	2022	Pubmed (Endocrine society, Oxford Academic, https://pubmed.ncbi.nlm.nih.gov/34871353/,zuletzt accessed 24.07.2025)	NA	FDA approved	Abstract
18	Dual Series Biochemistry (Rassow et al. 2022)	30%	2022	Textbook (Joachim Rassow, Karin Hauser, Roland Netzker, Rainer Deutzmann: Biochemie(= Duale Reihe), 5th edition, Stuttgart, Thieme, 2022, p.591)			
19	German Medical Journal (Aerzteblatt.de 2022)	A third	2022	Journal (https://www.aerzteblatt.de/nachrichten/134165/Neue-Rezeptorindungsstellen-fuer-GPCR-gerichtete-Medikamente-entdeckt, last accessed 24.07.2025)		Prescripted drugs	Article
20	(Miettinen et al. 2022)	34%	2022	Nature communications (https://www.nature.com/articles/s41467-022-31357-6, last accessed 24.07.2025)	1. cited A.S. Hauser, M.M. Attwood, M. Rask-Andersen, H.B. Schioth, D.E. Gloriam Trends in GPCR drug discovery: new agents, targets and indications Nat Rev Drug Discov, 16 (2017), pp. 829–842, 2. cited Sriram, K., and Insel, P.A. (2018). G Protein-Coupled Receptors as Targets for Approved Drugs: How Many Targets and How Many Drugs? Mol. Pharmacol. 93, 251–258. DOI: 10.1124/mol.117.111062.	FDA approved	Discussion
21	(Bielczyk-Maczynska et al. 2022)	34%	2022	Nature communications (https://www.nature.com/articles/s41467-022-35069-9, last accessed 24.07.2025)	cited A.S. Hauser, M.M. Attwood, M. Rask-Andersen, H.B. Schioth, D.E. Gloriam Trends in GPCR drug discovery: new agents, targets and indications Nat Rev Drug Discov, 16 (2017), pp. 829–842	Current FDA approved	Introduction
22	(Schulz et al. 2022)	35%	2022	Nature (https://www.nature.com/articles/s41467-022-32390-1#citeas, last accessed 24.07.2025)	1. cited Sriram, K., and Insel, P.A. (2018). G Protein-Coupled Receptors as Targets for Approved Drugs: How Many Targets and How Many Drugs? Mol. Pharmacol. 93, 251–258. DOI: 10.1124/mol.117.111062. 2. cited Tang, X., Wang, Y., Li, D., Luo, J. & Liu, M. Orphan G protein-coupled receptors (GPCRs): biological functions and potential drug targets. Acta Pharmacologica Sin. 33, 363–371 (2012)(https://www.nature.com/articles/aps2011210#ref-CR7)	FDA approved	Abstract
23	(Bean et al. 2022)	A third	2022	Nature (https://www.nature.com/articles/s41467-022-30570-7, last accessed 24.07.2025)	cited A.S. Hauser, M.M. Attwood, M. Rask-Andersen, H.B. Schioth, D.E. Gloriam Trends in GPCR drug discovery: new agents, targets and indications Nat Rev Drug Discov, 16 (2017), pp. 829–842	Current FDA approved	Introduction
24	(Watkins and Orlandi 2021)	Significant number	2021	Pubmed (british pharmacological society, https://pubmed.ncbi.nlm.nih.gov/33784795/, last accessed 24.07.2025)	1. cited A.S. Hauser, M.M. Attwood, M. Rask-Andersen, H.B. Schioth, D.E. Gloriam Trends in GPCR drug discovery: new agents, targets and indications Nat Rev Drug Discov, 16 (2017), pp. 829–842, 2. cited Sriram, K., and Insel, P.A. (2018). G Protein-Coupled Receptors as Targets for Approved Drugs: How Many Targets and How Many Drugs? Mol. Pharmacol. 93, 251–258. DOI: 10.1124/mol.117.111062.	FDA approved	Abstract
25	(Laschet and Hanson 2021)	30%	2021	Pubmed (G-Protein-Coupled Receptor Sceening Assasy Springer, https://pubmed.ncbi.nlm.nih.gov/34085267/, last accessed 24.07.2025)	1.cited A.S. Hauser, M.M. Attwood, M. Rask-Andersen, H.B. Schioth, D.E. Gloriam Trends in GPCR drug discovery: new agents, targets and indications Nat Rev Drug Discov, 16 (2017), pp. 829–842, 2. https://pubmed.ncbi.nlm.nih.gov/34085267/, 2. cited Sriram, K., and Insel, P.A. (2018). G Protein-Coupled Receptors as Targets for Approved Drugs: How Many Targets and How Many Drugs? Mol. Pharmacol. 93, 251–258. DOI: 10.1124/mol.117.111062.	Current FDA approved	Abstract
26	(Wang et al. 2021)	30–40%	2021	Pubmed (ScienceDirect, https://pubmed.ncbi.nlm.nih.gov/33338473/, last accessed 24.07.2025)	cited Lagerström, M., Schiöth, H. Structural diversity of G protein-coupled receptors and significance for drug discovery. Nat Rev Drug Discov 7, 339–357 (2008). https://doi.org/10.1038/nrd2518 https://www.nature.com/articles/nrd2518	Therapeutic drugs	Abstract, Introduction
27	(Wu et al. 2021)	40–50%	2021	Pubmed (IEEE/ACM, https://pubmed.ncbi.nlm.nih.gov/32750885/, last accessed 24.07.2025)	cited A. S. Hauser, et al., “Pharmacogenomics of GPCR drug targets,” Cell, vol. 172, no. 1–2, pp. 41–54, 2018	All drugs	Abstract
28	University of Würzburg (Schihada 2021)	34%%	2021	Website/Dissertation (https://opus.bibliothek.uni-wuerzburg.de/frontdoor/index/index/docId/17541, last accessed 24.07.2025)	1.cited A.S. Hauser, M.M. Attwood, M. Rask-Andersen, H.B. Schioth, D.E. Gloriam Trends in GPCR drug discovery: new agents, targets and indications Nat Rev Drug Discov, 16 (2017), pp. 829–842, 2.cited Sriram, K., and Insel, P.A. (2018). G Protein-Coupled Receptors as Targets for Approved Drugs: How Many Targets and How Many Drugs? Mol. Pharmacol. 93, 251–258. DOI: 10.1124/mol.117.111062.	FDA 2017	Introduction
29	(Kooistra et al. 2021)	34%	2021	Oxford Academic (https://academic.oup.com/nar/article/49/D1/D335/6018432, last accessed 24.07.2025)	cited A.S. Hauser, M.M. Attwood, M. Rask-Andersen, H.B. Schioth, D.E. Gloriam Trends in GPCR drug discovery: new agents, targets and indications Nat Rev Drug Discov, 16 (2017), pp. 829–842	Marketed drugs	Introduction
30	(Mantas et al. 2022)	A third	2022	Nature (https://www.nature.com/articles/s41380-021-01040-1, last accessed 24.07.2025)	cited A.S. Hauser, M.M. Attwood, M. Rask-Andersen, H.B. Schioth, D.E. Gloriam Trends in GPCR drug discovery: new agents, targets and indications Nat Rev Drug Discov, 16 (2017), pp. 829–842	FDA approved	Main
31	(Cornwell and Feigin 2020)	35%	2020	Pubmed (Cellpress, https://pubmed.ncbi.nlm.nih.gov/33198923/, last accessed 24.07.2025)	cited Sriram, K., and Insel, P.A. (2018). G Protein-Coupled Receptors as Targets for Approved Drugs: How Many Targets and How Many Drugs? Mol. Pharmacol. 93, 251–258. DOI: 10.1124/mol.117.111062.	FDA approved	Abstract, Introduction
32	(Shchepinova et al. 2020)	30%	2020	Pubmed (ScienceDirect, https://pubmed.ncbi.nlm.nih.gov/32446179/, last accessed 24.07.2025)	NA	Marketed drugs	Abstract, Introduction
33	(Li et al. 2020)	30%	2020	Pubmed (Journal of medicinal chemistry, https://pubmed.ncbi.nlm.nih.gov/31841625/, last accessed 24.07.2025)	cited Hopkins, A., Groom, C. The druggable genome. Nat Rev Drug Discov 1, 727–730 (2002). https://doi.org/10.1038/nrd892	Drugs in use	Abstract
34	(Perini et al. 2020)	30–50%	2020	Pubmed (ScienceDirect, https://pubmed.ncbi.nlm.nih.gov/32173557/, last accessed 24.07.2025)	NA	Marketed drugs	Abstract
35	(Kimura et al. 2020)	30–40%	2020	Pubmed (MDPI, https://pubmed.ncbi.nlm.nih.gov/33076386/, last accessed 24.07.2025)	cited Lagerström, M., Schiöth, H. Structural diversity of G protein-coupled receptors and significance for drug discovery. Nat Rev Drug Discov 7, 339–357 (2008). https://www.nature.com/articles/nrd2518	Drugs in use	Abstract, Introduction
36	(Kobayashi, Y. et al. 2020)	30%	2020	Pubmed (Oxford Academic, https://pubmed.ncbi.nlm.nih.gov/32627821/, last accessed 24.07.2025)	NA	Approved drugs	Abstract
37	University of Marburg, Jelinek, V: Studies on the selective recognition of G-proteins by GPCRs and their downstream activation using mutagenesis studies	30%	2020	Website (University. Marburg, https://archiv.ub.uni-marburg.de/ubfind/Record/urn:nbn:de:hebis:04-z2020-0530/Description#tabnav, last accessed 24.07.2025)	NA	Approved drugs	Summary
38	Mutschler drug effects (Geisslinger et al. 2020)	35%	2020	Textbook (Geisslinger, Menzel, Gudermann, Hinz, Ruth: Mutschler Arzneimittelwirkungen Pharmakologie Klinische Pharmakologie Toxikologie, 11th edition, Stuttgart, Wissenschaftliche Verlagsgesellschaft Stuttgart, 2020, p.93)	NA		
39	Sunny Al-Shamma, n.d. President and CEO of Beacon Discovery in nature research	30%	2020	Nature research (https://www.nature.com/articles/d43747-020-01094-0, last accessed 24.07.2025)	Direct quote from person	Approved drugs	Direct quote
40	(Congreve et al. 2020)	34%	2020	CellPress (https://www.sciencedirect.com/science/article/pii/S0092867420302658, last accessed 24.07.2025)	cited R. Santos, O. Ursu, A. Gaulton, A. P. Bento, R. S. Donadi, C. G. Bologa, A. Karlsson, B. al-Lazikani, A. Hersey, T. I. Oprea, J. P. Overington, A comprehensive map of molecular drug targets. Nat. Rev. Drug Discov. 16, 19–34 (2017). Cited R. C. Steve	Small molecule drugs	Main
41	(Ribeiro-Oliveira et al. 2019)	30%	2019	Pubmed (ScienceDirect, https://pubmed.ncbi.nlm.nih.gov/31520747/, last accessed 24.07.2025)	NA	Marketed drugs	Abstract
42	Essential Cell Biology (Alberts et al. 2019)	A third	2019	Textbook (Alberts, Hopkin, Johnson, Morgan, Raff, Roberts, Walter: Essential Cell Biology. 5th edition, Norton, 2019, p.545)			
43	Medical University of Vienna (Gruber, Christian, 2019)	30%	2019	Website (University of Vienna, https://www.meduniwien.ac.at/web/ueber-uns/news/detailseite/2019/news-im-dezember-2020/peptidforschung/, last accessed 24.07.2025)	Direct quote from person	All drugs	Direct quote
44	(Benredjem et al. 2019)	30%	2019	Nature communications (https://www.nature.com/articles/s41467-019-11875-6, last accessed 24.07.2025)	cited Lafferty-Whyte, K., Mormeneo, D. & del Fresno Marimon, M. Opportunities and challenges of the 2016 target landscape. Nat Rev Drug Discov 16, 10–11 (2017). https://doi.org/10.1038/nrd.2016.263	FDA approved	Introduction
45	(Nature Chemical Biology, (, 2019) Beyond or off.)	34%	2019	Nature Chemical Biology (https://www.nature.com/articles/s41589-018-0202-5#rightslink, last accessed 24.07.2025)	NA	FDA approved	Main
46	(Syu et al. 2019)	34%	2019	Nature (https://www.nature.com/articles/s41467-019-09938-9, last accessed 24.07.2025)	1.cited A.S. Hauser, M.M. Attwood, M. Rask-Andersen, H.B. Schioth, D.E. Gloriam Trends in GPCR drug discovery: new agents, targets and indications Nat Rev Drug Discov, 16 (2017), pp. 829–842, 2.cited Sriram, K., and Insel, P.A. (2018). G Protein-Coupled Receptors as Targets for Approved Drugs: How Many Targets and How Many Drugs? Mol. Pharmacol. 93, 251–258. 10.1124/mol.117.111062.	FDA	Introduction
47	(Ayoub 2018)	30–40%	2018	Pubmed (ScienceDirect, https://pubmed.ncbi.nlm.nih.gov/29522725/, last accessed 24.07.2025)	cited A.S. Hauser, M.M. Attwood, M. Rask-Andersen, H.B. Schioth, D.E. Gloriam Trends in GPCR drug discovery: new agents, targets and indications Nat Rev Drug Discov, 16 (2017), pp. 829–842	Approved drugs	Abstract
48	(Calebiro and Godbole 2018)	30–50%	2018	Pubmed (ScienceDirect, https://pubmed.ncbi.nlm.nih.gov/29678288/, last accessed 24.07.2025)	NA	Prescribted drugs	Abstract
49	(Villanueva 2018)	30%	2018	Nature reviews (https://www.nature.com/articles/nrd.2018.13#citeas, last accessed 24.07.2025)	NA	FDA approved	Abstract
50	(Martemyanov and Garcia-Marcos 2018)	More than 30%	2018	Journal (Journal of Biological Chemistry, https://www.jbc.org/article/S0021-9258(20)39231-0/pdf, last accessed 24.07.2025)	cited Sriram, K., and Insel, P.A. (2018). G Protein-Coupled Receptors as Targets for Approved Drugs: How Many Targets and How Many Drugs? Mol. Pharmacol. 93, 251–258. DOI: 10.1124/mol.117.111062.	FDA approved	Main
51	(Kono et al. 2017)	30%	2017	Nature (https://www.nature.com/articles/s41467-017-01340-7, last accessed 24.07.2025)	1. cited Roth, B. L. & Kroeze, W. K. Integrated approaches for genome-wide interrogation of the druggable non-olfactory g protein-coupled receptor superfamily. J. Biol. Chem. 290, 19,471–19,477 (2015) 2. cited Hopkins, A., Groom, C. The druggable genome. Nat Rev Drug Discov 1, 727–730 (2002). https://doi.org/10.1038/nrd892	Approved drugs	Introduction
52	(Liu et al. 2016)	30–40%	2016	Pubmed (ScienceDirect, https://pubmed.ncbi.nlm.nih.gov/26920251/, last accessed 24.07.2025)	NA	Marketed drugs	Abstract
53	An Audiance with Michael. (, 2016)	40%	2016	Nature drug discovery (https://www.nature.com/articles/nrd.2016.12, last accessed 24.07.2025)	probably cited Hopkins, A., Groom, C. The druggable genome. Nat Rev Drug Discov 1, 727–730 (2002). https://doi.org/10.1038/nrd892	All drugs	Interview
54	(Duc et al. 2015)	40%	2015	Pubmed (ScienceDirect, https://pubmed.ncbi.nlm.nih.gov/25981300/, last accessed 24.07.2025)	NA	Marketed drugs	Abstract, Introduction
55	(Romanova and Sweedler 2015)	30%	2015	Pubmed (Cellpress, https://pubmed.ncbi.nlm.nih.gov/26143240/, last updated 24.07.2025)	NA	Commercial drugs	Abstract
56	(Ghosh et al. 2015)	The half	2015	Nature (https://www.nature.com/articles/nrm3933, last accessed 24.07.2025)	cited Ma, P. & Zemmel, R. Value of novelty? Nature Rev. Drug Discov. 1, 571–572 (2002)	Approved drugs	
57	(Bermudez Sasso 2015)	30%	2015	Dissertation (https://refubium.fu-berlin.de/handle/fub188/5859?show=full, last accessed 24.07.2025)	cited Overington, J.P., B. Al-Lazikani, and A.L. Hopkins, Opinion—How many drug targets are there? Nat. Rev. Drug Discovery, 2006	All drugs	Introduction
58	(Brogi et al. 2014)	30%	2014	Pubmed (Frontiers, https://pubmed.ncbi.nlm.nih.gov/25506327/, last accessed 24.07.2025)	NA	Approved drugs	Abstract
59	(Urs et al. 2014)	30%	2014	Pubmed (ScienceDirect, https://pubmed.ncbi.nlm.nih.gov/24680431/, last accessed 24.07.2025)	NA	Marketed drugs	Abstract
60	(Solinski et al. 2014)	30%	2014	Pubmed (Aspet, https://pubmed.ncbi.nlm.nih.gov/24867890/, last accessed 24.07.2025)	NA	Modern drugs target	Abstract
61	(Stevens et al. 2013)	30–40%	2013	Pubmed (Nature, https://pubmed.ncbi.nlm.nih.gov/23237917/, last accessed 01.06.2025)	cited Wise A, Gearing K, Rees S. Target validation of G-protein coupled receptors. Drug Discov Today. 2002; 7:235–246.	Marketed drugs	Abstract
62	(Katritch et al. 2012)	40%	2012	Pubmed (Cellpress https://pubmed.ncbi.nlm.nih.gov/22032986/, last accessed 02.06.2025)	cited Wise A, Gearing K, Rees S. Target validation of G-protein coupled receptors. Drug Discov Today. 2002;7:235–246. doi: 10.1016/s1359-6446(01)02131-6.	Approved drugs	Introduction
63	(Martins et al. 2012)	30%	2012	Pubmed (Cellpress, https://pubmed.ncbi.nlm.nih.gov/22921755/, last accessed 24.07.2025)	NA	Marketed drugs	Abstract
64	(Tang et al. 2012)	36%	2012	Nature (https://www.nature.com/articles/aps2011210, last accessed 24.07.2025)	cited Rask-Andersen M, Almen MS, Schioth HB. Trends in the exploitation of novel drug targets. Nat Rev Drug Discov 2011; 10: 579–90	Marketed drugs	Abstract, Introduction
65	(Gruber, Christian W. et al. 2010)	30–50%	2010	Pubmed (https://pubmed.ncbi.nlm.nih.gov/20687879/, last accessed 24.07.2025)	1. cited Jimonet P, Jaeger R. Strategies for designing GPCR-focused libraries and screening sets. Curr Opin Drug Discov Develop. 2004;7:325–33 2. cited Klabunde T, Hessler G. Drug design strategies for targeting G-protein-coupled receptors. ChemBioChem. 2002;3:928–44. doi: 10.1002/1439-7633(20021004)3:10<928::AID-CBIC928>3.0.CO;2-5. 3. cited Tyndall JDA, Pfeiffer B, Abbenante G, et al. Over One Hundred Peptide-Activated G Protein-Coupled Receptors Recognize Ligands with Turn Structure. Chem Rev. 2005;105:793–826. doi: 10.1021/cr040689g.	Marketed drugs	abstract, introduction
66	(Simon et al. n.d., Sep 17, 2008)	30%	2008	Journal (https://www.thieme-connect.com/products/ejournals/abstract/10.1055/s-0028-1089702, last accessed 24.07.2025)	NA	All drugs	Introduction
67	(Ja and Roberts 2005)	More than half	2005	Pubmed (Cellpress, https://pubmed.ncbi.nlm.nih.gov/15950876/, last accessed 24.07.2025)	NA	Prescribted drugs	Abstract
68	(Wise et al. 2002)	30%	2002	Pubmed (ScienceDirect) https://pubmed.ncbi.nlm.nih.gov/11839521/, last accessed 02.06.2025)	cited J. Drews Drug discovery: a historical perspective Science, 287 (2000), pp. 1960–1964	Marketed drugs	Introduction
69	(Ma and Zemmel 2002)	50%	2002	Pubmed (Nature https://pubmed.ncbi.nlm.nih.gov/12402497/, last accessed 02.06.2025)	NA	All drugs	Abstract

## Material and methods

In this paper, the number/percentage of newly approved GPCR drugs in Germany in the last 37 years is compared with the total number of approvals in this period (Table S1 and Table 2). The book series"Neue Arzneimittel"by Fricke and Klaus and the Arzneiverordnungs-Report (AVR) by Ludwig et al. were used as sources for the newly approved drugs (Table S1 and Table 2). During this period, a total of 1,110 drugs were approved in Germany according to these sources. All newly approved drugs in Germany during this period were listed in excel and assigned to a primary mechanism of action. The number of categories of mechanisms of action was limited to 30.

**Table 2:** Brief presentation of the sources used for assigning the mechanism of action of drugs and the number of GPCR drugs

Num-ber	Source	Author/Publisher	Used years	Short description	Weblink
1	Neue Arzneimittel	Uwe Fricke/Wolfgang Klaus	1987–1997	Detailed overview of newly approved drugs in the respective year. In later years also including the therapeutic value	https://www.deutscher-apotheker-verlag.de/Neue-Arzneimittel-Band-22/9783804737204, last accessed 02.08.2025
2	Arzneiverordnungsreport (AVR)	Wolf-Dieter Ludwig, Bernd Mühlbauer and Roland Seifert	1998–2024	Reference book and overview of the current pharmaceutical market. Detailed information on the individual drugs. A separate chapter on new drugs in the respective year	https://link.springer.com/book/10.1007/978-3-662-70594-0, last accessed 02.08.2025
3	WHO Model of Essential Medicine	WHO Expert Committee	1999/2000, 2002, 2023	Drug substances of outstanding importance on the current pharmaceutical market	https://www.who.int/groups/expert-committee-on-selection-and-use-of-essential-medicines/essential-medicines-lists, last accessed 02.08.2025
4	IMPP	Institut für medizinische und pharmazeutische Prüfungsfragen	2021, 2024	Drug list based on the Gegenstandskatalog (subject catalog) of the examination (2nd state examination) in medical studies	https://www.mhh.de/fileadmin/mhh/zentrum-pharmakologie-toxikologie/download/IMPP-Arzneistoffliste.pdf, last accessed 02.08.2025
5	Rote Liste	Rote Liste Service GmbH	2002,2023	Drug directory containing the current preparations of companies organized in the Federal Association of the Pharmaceutical Industry in Germany	https://www.rote-liste.de, last accessed 02.08.2025

In this analysis, “drug” refers to a newly approved active substance. This also includes monoclonal antibodies (“biologicals”), contrast agents and other diagnostic substances, as well as vaccines (Covid 19 vaccines excluded). The primary mechanism of action of each drug was assigned. The assignment of a drug to a drug target is often ambiguous. For mechanistic assignment we used the AVR, “Neue Arzneimittel”, the FDA and websites such as go.drugbank.com, guidetopharmacology.org and gelbe-liste.de as a guide. In cases of ambiguity, we have opted for the supposedly primary target. In cases of doubt, the specific mechanism of action was listed and then classified under “other”.

To enable a comparison with drugs that have been approved for some time and the German pharmaceutical market in general, the Rote Liste (Red List) from 2002 and 2023 was analyzed (Table S1 and Table 2). The Rote Liste (Red List) contains comprehensive information on drugs marketed in Germany. This list of drugs is published annually in book form and every six months in electronic form (https://www.rote-liste.de/ueber-rote-liste, last accessed on 16.06.2025). Another source is the list of the Institute for Medical and Pharmaceutical Exam Questions (Table S1 and Table 2). The drugs that act on GPCRs in these sources are then related to the total number of drugs.

**Fig. 1:**
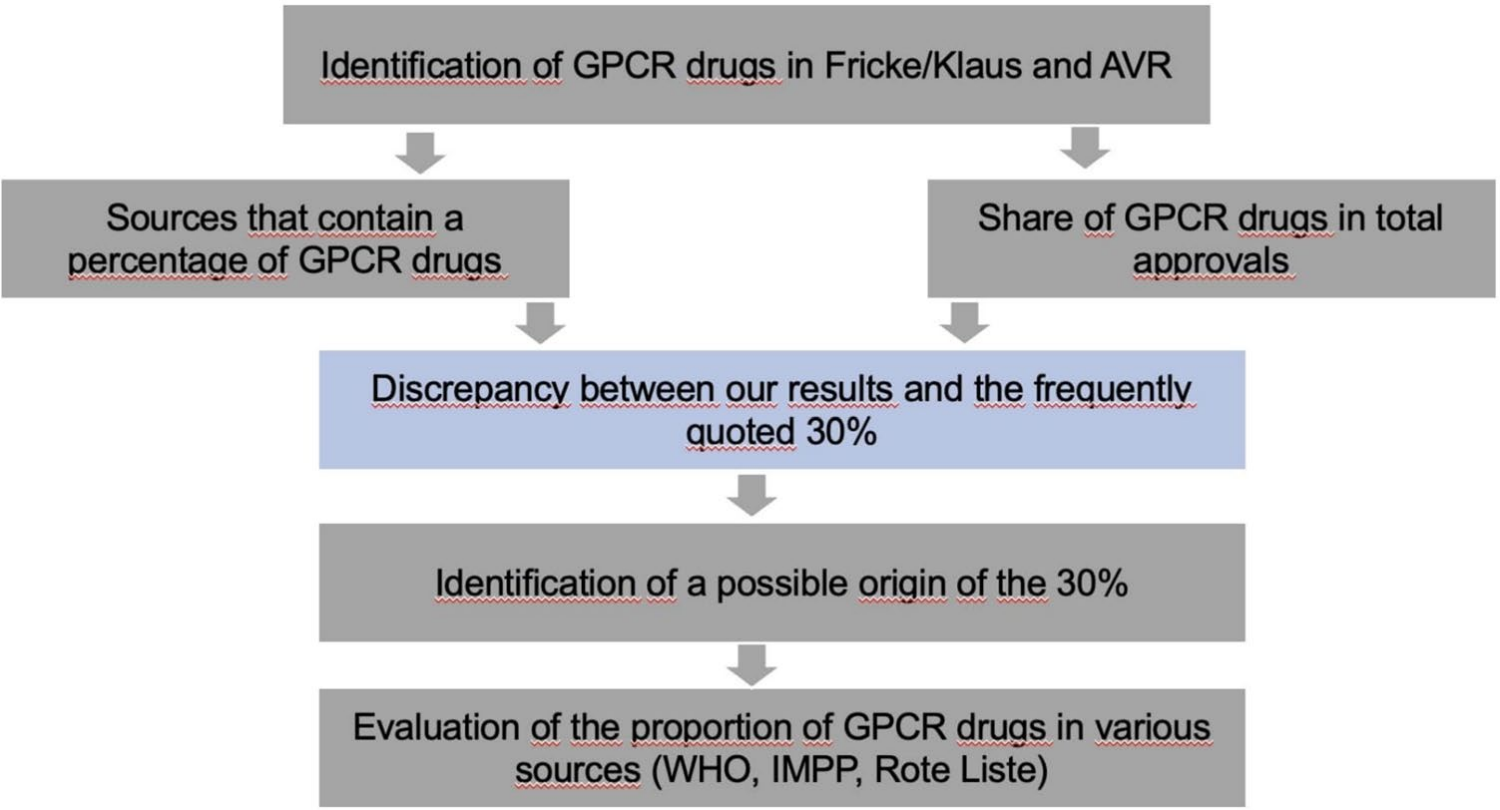
Flowchart of the methodological approach. The starting point was the number of GPCR drugs in"Neue Arzneimittel"and the AVR. This was followed by a comparison with sources that cited percentages, a search for the origin of these and a comparison with other sources

To provide an international perspective the WHO Model List of Essential Medicines from several years was analyzed. The edition from the turn of the millennium 1999/2000 was used, as well as the year 2002 and the year 2023. The year 2002 was selected because a paper from 2002 was identified as the probable starting point of the statement that 30% of all drugs act on GPCRs (Hopkins and Groom 2002). The WHO List of Essential Medicines has been published since 1977 and specifically for children since 2007. The list is updated every 2 years by a committee of experts. The experts come from various disciplines and countries (https://www.who.int/groups/expert-committee-on-selection-and-use-of-essential-medicines/essential-medicines-lists, last accessed 13.06.2025). Figure 1 shows an overview of the methodology. To support our data with another international source, we analyzed the drugs newly approved by the FDA between 2018 and 2023.

## Methods (Fig. 1 and Table 3)

We identified seven studies in which databases were analyzed to assess the percentage of GPCR drugs (Table 3). The remaining papers merely cited the number of GPCR drugs but did not always cite a source. In some studies, it was not stated which drugs the 30% are referred to (see Table 1).

**Table 3:** Sources providing the percentage of GPCR drugs based on the analysis of databases

Number	Authors	Share of GPCR drugs	Year	Drugs	Source	Cited (16.06.2025)
1	(Lorente et al. 2025)	36%	2025	DrugBank and Open Targets—> 516 GPCR drugs	Pubmed (Nature, https://pubmed.ncbi.nlm.nih.gov/40033110/, last accessed 16.06.2025)	3
2	(Hauser et al. 2017)	34%	2017	GPCR: 481 FDA approved drugs and 320 agents in clinical trials	Pubmed (Nature, https://pubmed.ncbi.nlm.nih.gov/29075003/, last accessed 25.05.2025)	1843
3	(Sriram and Insel 2018)	35%	2018	chEMBL, Guide to OHARMACOLOGY, DrugBank—> ∼1950 drugs EMA and FDA drugs	Pubmed (Molecular pharmacology, https://pubmed.ncbi.nlm.nih.gov/29298813/, last accessed 25.05.2025)	858
4	(Santos et al. 2017)	33%%	2017	ChEMBL, DrugCentral and canSAR—> 1578 FDA approved drugs	https://www.nature.com/articles/nrd.2016.230, last accessed 02.06.2025)	1579
5	(Roth and Kroeze 2015)	9 out of 41—> ∼ 22%	2015	41 FDA approved drugs in 2014	https://www.jbc.org/article/S0021-9258(20)42207-0/fulltext, last accessed 02.06.2025	69
6	(Rask-Andersen et al. 2011)	36%	2011	1542 FDA approved drugs	https://pubmed.ncbi.nlm.nih.gov/21804595/, last accessed 02.06.2025	677
7	(Overington et al. 2006)	27%	2006	US FDA’s Orange Book—> 1357 FDA drugs and the Center for Biologics Evaluation and Research (CBER) website	https://pubmed-1ncbi-1nlm-1nih-1gov-1v67xgbwn008a.mhh.hh-han.com/17139284/, last accessed 02.06.2025	2831

## Results and discussion

### Share of GPCR drugs in the total number of new drugs in Germany

**Fig. 2:**
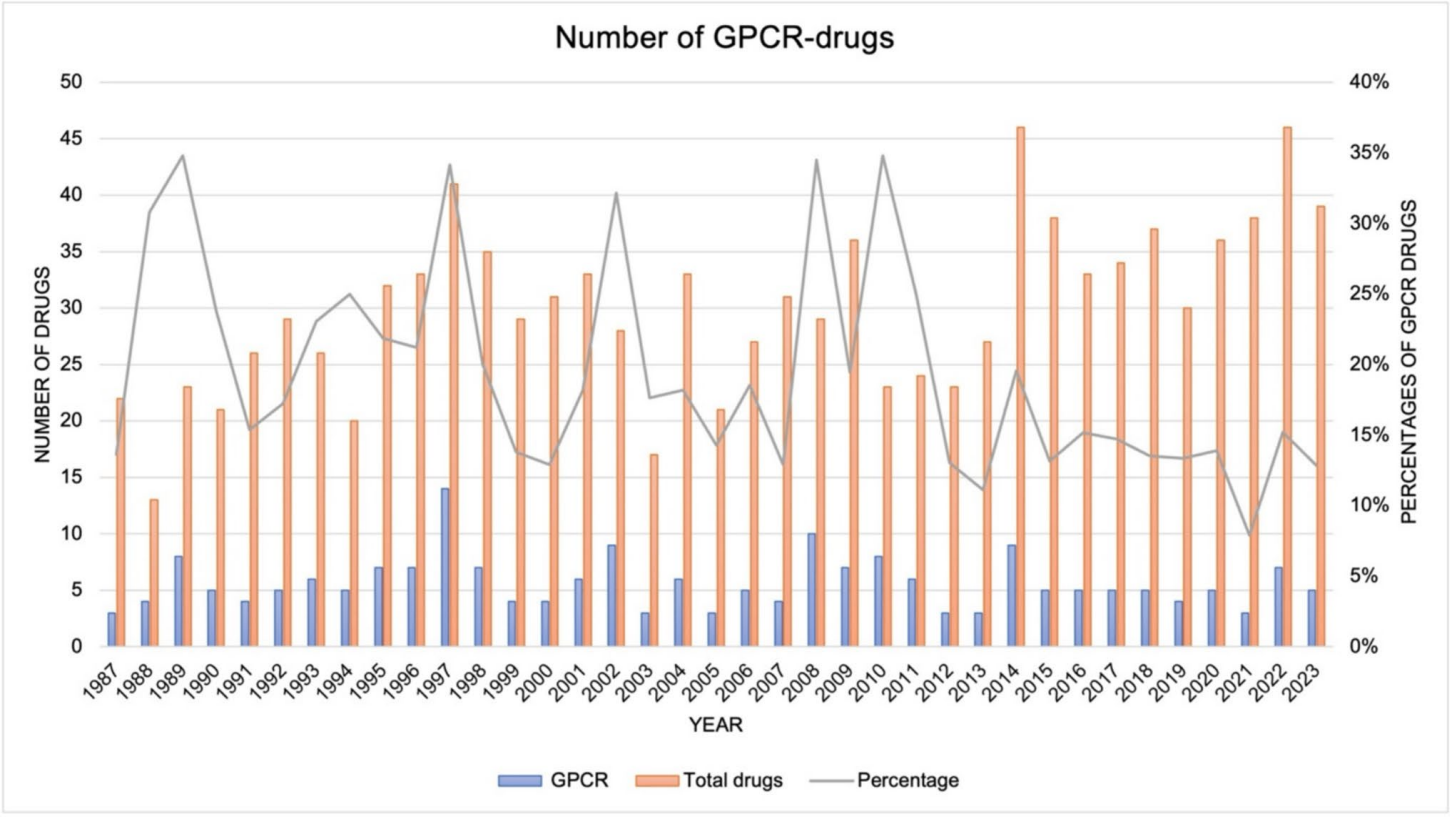
Overview of the total drug approvals per year and the GPCR drugs starting from 1987 to 2023. The orange bars show the total number of drugs per year. The blue bars show the number of GPCR drugs per year. The grey line shows the percentages of GPCR drugs per year from 1987 to 2023

The number of newly approved drugs in Germany between 1987–2023 fluctuated within a range of 13 to 46 (Fig. 2). The number of new GPCR drugs varied between 3–14 per year with multiple peaks.

In 1989,1997 and 2008, the share of GPCR drugs was over 34%, making this the years with the highest share. In 2021, the share was the lowest at 7.9%. Between 2013 and 2023, the percentage of GPCR drugs ranged between 8 and 20%.

### Comparison of the actual percentages with the 30% value

**Fig. 3:**
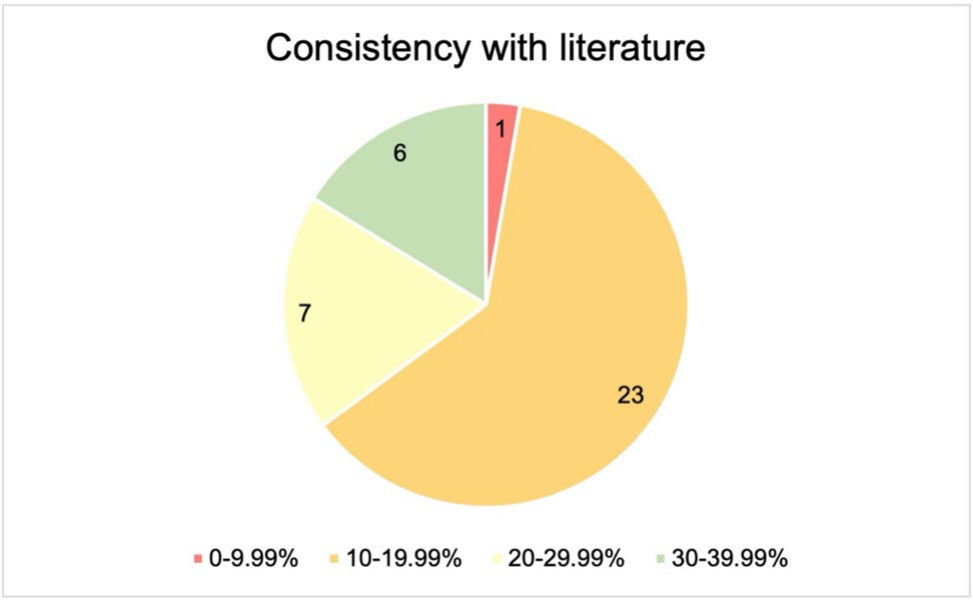
Coding of the proportion of GPCR drugs approved each year according to traffic light system

In Fig. 3, the percentages of annual GPCR drug approvals are divided into areas. These areas are assigned colors according to a traffic light system. The reference value for green was the frequently cited 30%. The years in the 20–29.99% range are coded yellow. The range 10–19.99% is orange. The years in which less than 10% of the drugs act on GPCRs are coded red. Only in six years the proportion of GPCR drugs exceeded 30%. Seven years showed a proportion of 20%−29.99%. In most years (23) the proportion was between 10–19.99%. In 2021 the proportion of GPCR drugs was less than 10%.

### Possible origin of the 30% value

**Fig. 4:**
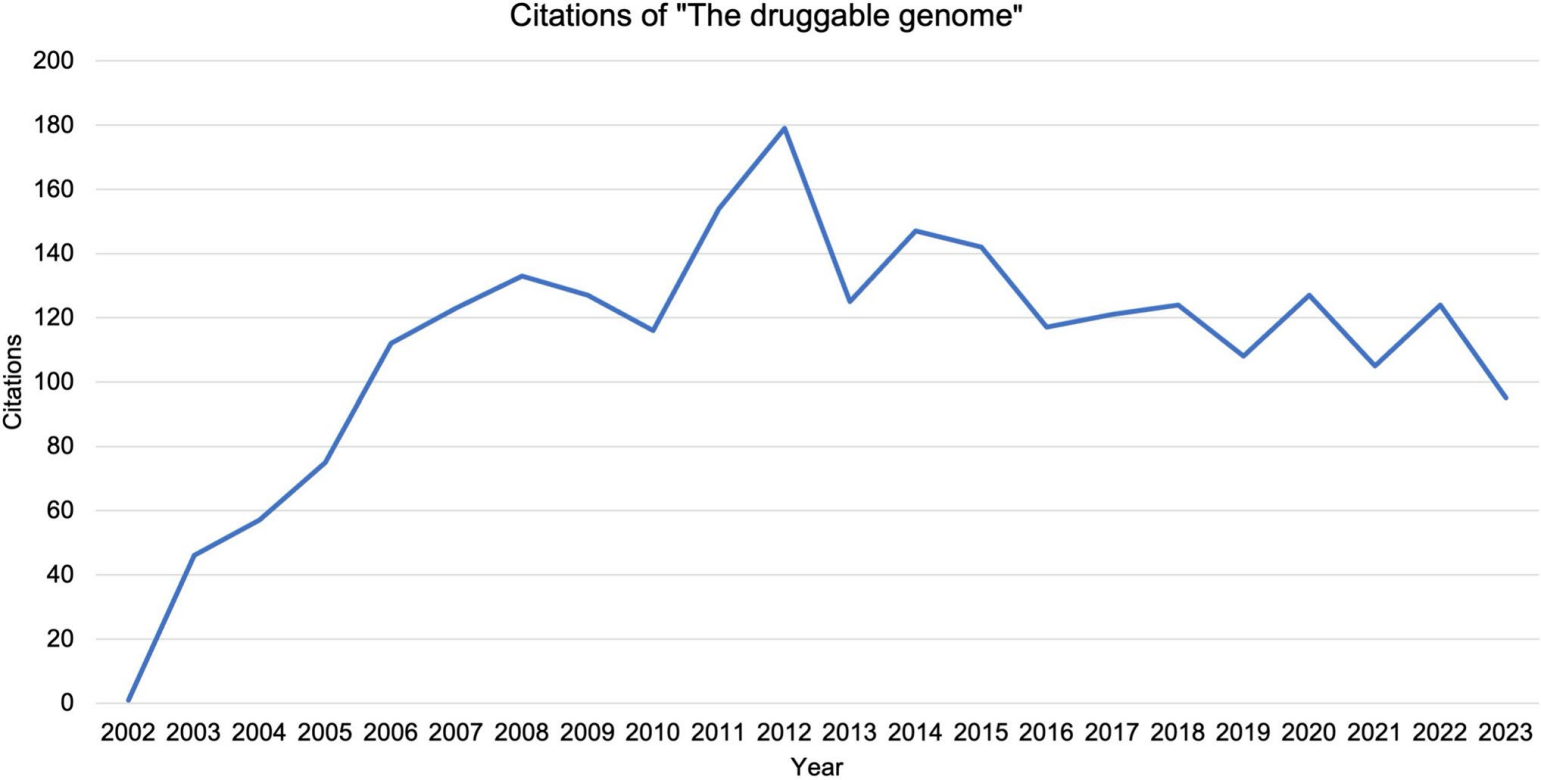
Citations of "The druggable genome" from Hopkins and Groom. Sorted by year, starting with 2002

GPCRs were known as important drug targets already in the 1990s. However, papers published in this decade usually did not provide exact percentages (Barak et al. 1997), (Tarasova et al. 1999). On September 1, 2002, the paper "The druggable genome" by Andrew L. Hopkins and Colin R. Groom was published in Nature Reviews Drug Discovery (Hopkins and Groom 2002). The paper was in the 98th percentile of articles of a similar age. According to the journals'internal metrics, the article was accessed approximately 37,000 times and cited almost 3000 times. (https://www.nature.com/articles/nrd892/metrics, last accessed 24.07.2025). Figure 4 shows an analysis of the citations of the paper in the Web of Science database (Clarivate). As of September 2024, the work has been cited 2519 times according to the database. The number of citations increased almost linearly up to 2008. The work was cited most frequently in 2012 (180). On average, the work was cited 111 times per year in the period 2002–2023.

The analysis by Hopkins and Groom reports that 25% of all experimental and marketed drugs act on GPCRs (see Table 4). 15% of the"druggable genome"are described as addressable via GPCRs and 30% of all drugs on the market at that time were reported to act on GPCRs (Hopkins and Groom 2002). Thus, three different percentages are given for GPCRs. While in Fig. 1a all drugs belonging to Lipinski's"rule-of-five"analysis are analyzed, it is unclear to which drugs Fig. 4 refers. Figure 4 makes the generalized statement that 30% of the drugs on the market act on GPCRs. It is not clear from the legend or the text to which country, period or drug list the figure refers. Based on this analysis, it can be assumed that the 30% value stems from Fig. 4 of the paper from Hopkins and Groom (2002) with the source data being at best elusive. But it appears that in later studies, the 30% value was welcomed by some authors in the field without asking further questions about the validity of the number.

**Table 4:** Analysis of the percentages of GPCR drugs in the paper 'The druggable genome' by Hopkins and Groom (2002). The data are compared with our results and the WHO List of Essential Medicines from the years 2023, 2002 and 1999/2000

Figure in"The druggable genome"	Legend	Percent GPCR	Meaning	Interpretation
Figure 1a Drug-target families	Gene-family distribution of the molecular targets of current rule-of-five-compliant experimental and marketed drugs	25%	25% of experimental and marketed drugs work on GPCRs	25% is slightly lower than the much publicized 30%, which is because experimental and not yet approved drugs are also included
Figure 1b Drug-target families	Gene-family distribution of the druggable genome	15%	GPCRs make up 15% of the druggable genome—> the part of the genome that can be addressed by drugs	15% is most consistent with our study and the WHO lists from 1999/2000, 2002 and 2023 (see SF1). Whereby the 15% in Hopkins'study represents the proportion of the druggable genome and our ≈ 17% refers to the proportion of drugs that act on GPCRs
Figure 4	Marketed small-molecule drug targets by biochemical class	30%	30% of all marketed drugs act on GPCRs	The 30% from this figure probably represents the origin of the spread of this number

### Comparison of the percentage of GPCR drugs in various sources

**Fig. 5:**
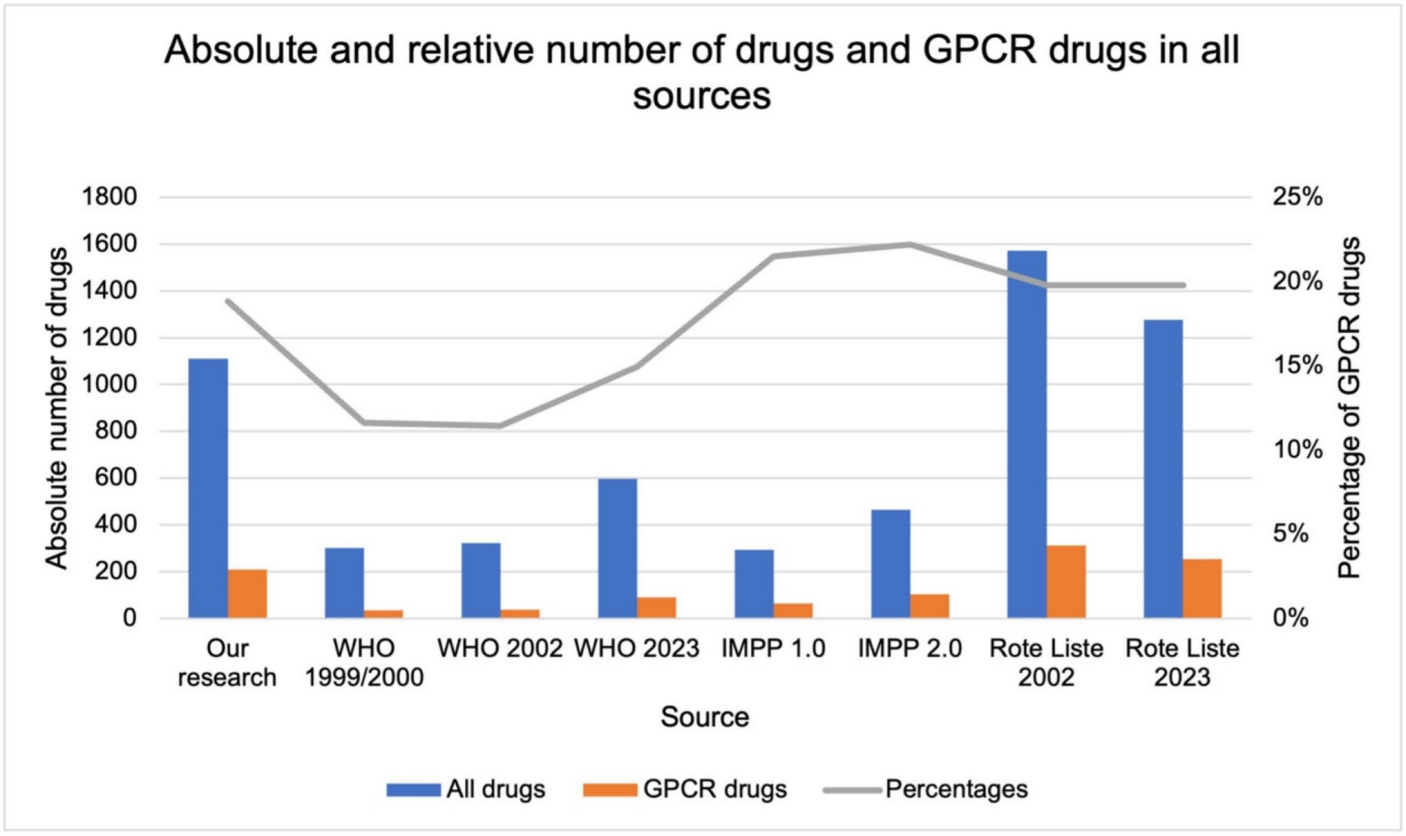
The blue bars show the absolute number of all drugs in the respective sources. The orange bars show the absolute number of GPCR drugs in the respective sources. The grey line shows the percentages of GPCR drugs. The complete drug lists are presented in Table S1

Of the 1110 newly approved drugs approved in Germany between 1987–2023, 209 drugs act on GPCRs (about 19%) (Fig. 5). The WHO list of Essential Medicines from 1999/2000 comprises 301 drugs, 35 of which act on GPCRs (about 12%). The WHO list from 2002 comprises 323 drugs, 37 of which act on GPCRs (about 11%). The WHO list from 2023 includes 596 drugs, 89 of which act on GPCRs (about 15%). In none of the WHO lists analyzed does the percentage come close to 30%. The Rote Liste (Red List) lists all marketed drugs from companies that are organized in the Bundesverband (Federal Association) of the Pharmaceutical Industry in Germany. In 2002, the list contained 1572 drugs, 311 of which are GPCR drugs (about 20%). In 2023, the Rote Liste comprised 1278 drugs, 253 of which are GPCR drugs (about 20%). The lists of the IMPP comes closest to 30%. In the version of 2021 (1.0) of the 293 drugs, 63 acts on GPCRs (about 22%). The updated version from 2024 (2.0) includes 464 drugs, 103 drugs act on GPCR (about 22%). However, this is not a list relevant for prescription of drugs in the clinic or practice but rather a drug list for teaching medical students. Thus, the list also includes drugs with “didactic” rather than clinical value. Just 11 GPCR occurred in all sources listed in Table S1, suggesting a small consensus on highly important GPCR drugs (Table 5).

**Table 5:** These 11 GPCR drugs were listed in all sources analyzed in Table S1. The drugs are sorted alphabetically

Number	Drug
1	Adrenaline
2	Atropine
3	Biperidene
4	Haloperidol
5	Ipratropium bromide
6	Morphine
7	Naloxone
8	Pilocarpine
9	Propranolol
10	Salbutamol
11	Timolol

### Comparing the FDA approved drugs 2018–2023 to our data

**Fig. 6:**
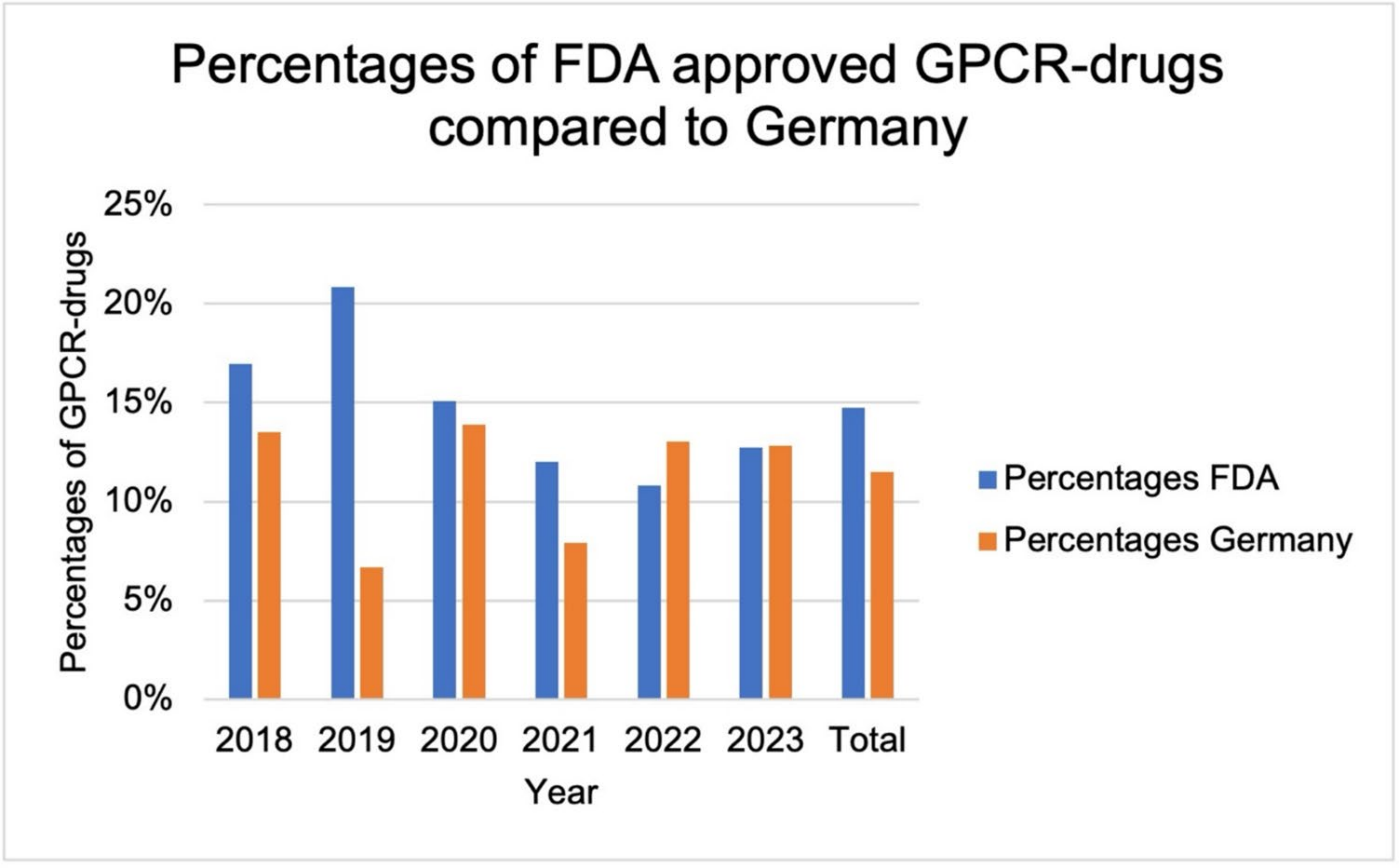
Percentages of GPCR-drugs FDA compared to Germany. The blue bar shows the percentages of newly approved GPCR- drugs by the FDA from 2018–2023 and in total for this period. The orange bar shows the percentages of newly approved GPCR-drugs in Germany from 2018–2023 and in total for this period

To establish a better reference to international data, the FDA-approved drugs from 2018–2023 were analyzed with regard to the absolute number and percentage of GPCR-drugs. 302 drugs were approved in this period. In these six years, between 59 (2018) and 37 (2022) drugs were approved. In 2019, the proportion of drugs that act on GPCRs was 20.8% (Fig. 6). The lowest proportion was 10.8% in 2022. On average, 14.5% of the drugs acted on GPCRs.

**Fig. 7:**
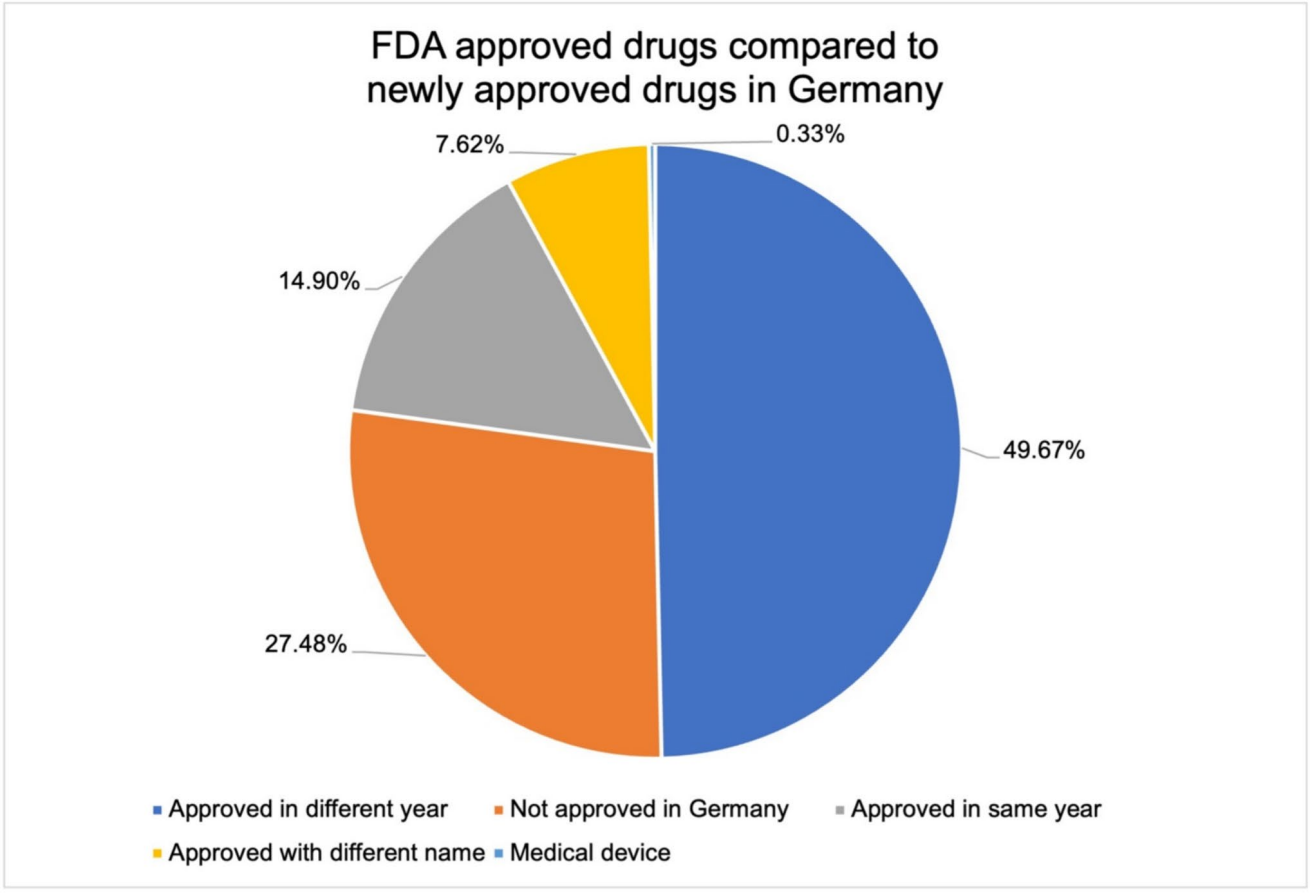
Comparison between dan FDA approved drugs 2018–2023 with the German pharmaceutical market. The blue area shows the proportion of drugs that were approved in a different year. The orange area shows the drugs that are not (yet) approved in Germany. The gray area shows the proportion of drugs that were approved in the same year. The yellow area shows the proportion of drugs that were approved under a different name. The gray area shows the drugs that were approved as medical device in Germany

2019 is a year with few GPCR drugs in Germany (13%) but the year with the most GPCR drugs from the FDA. To explain this difference, the drugs were compared in terms of their approval year and approval status. Figure 7 shows the analysis of FDA-approved drugs compared to Germany (see Table S1). Around 50% of the drugs were approved by the FDA in a different year than in Germany. This means that the drugs were also listed in the AVR in a different year and therefore in a different year in our analysis. Approx. 27% of the drugs were not (yet) approved in Germany at the current time (June 2025). Some drugs (8%) were approved in Germany under a different name. One drug has been approved by the FDA as a drug but is considered a medical device in Germany. These differences show how difficult it is to make a generally valid statement about the drug market.

### Mechanism of action of newly approved drugs in Germany

**Fig. 8:**
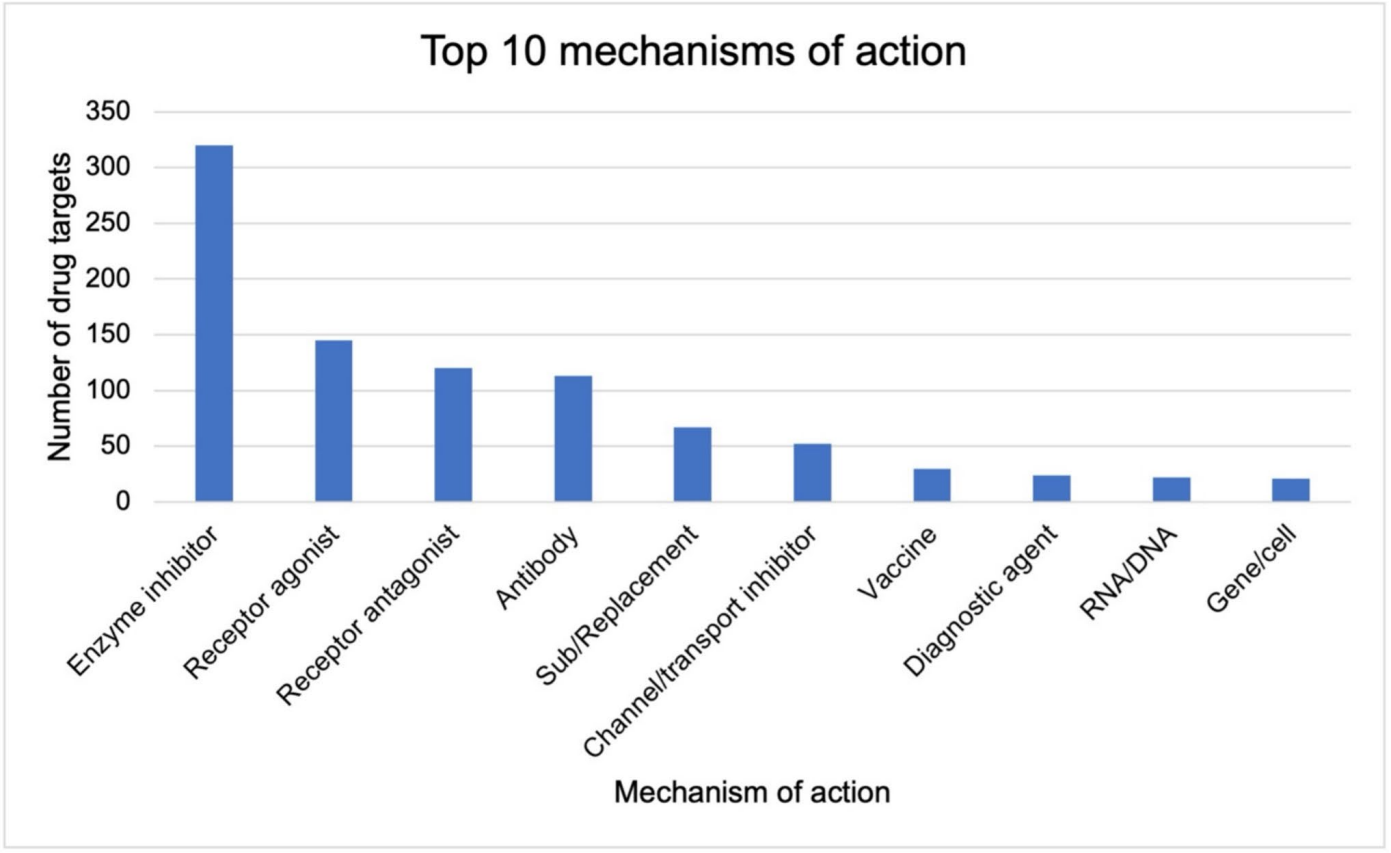
Leading 10 mechanisms of action of newly approved drugs in Germany from 1987–2023

30 different mechanisms of action were identified. Figure 8 shows the top 10 mechanisms of action in descending order. The most frequently addressed target structures are enzymes (324 times in total). Most of these drugs act as enzyme inhibitors (320). The second most common target structure is receptors. A total of 294 drugs act on a receptor, most of them as receptor agonists (145). Antibodies are an increasingly important mechanism of action. In the 37 years studied, a total of 113 drugs act via this mechanism. 10 drugs are antibody conjugates (not listed in top 10). The 67 drugs classified as supplements or replacements include hormones or vitamins, for example, but also recombinant drugs. 52 drugs act as channel or transporter inhibitors. Vaccines (30) follow in seventh place. Between 1987 and 2023, 30 different vaccines were approved, not including Covid-19 vaccines. The diagnostic agents (24) primarily include contrast media (20). They also include drugs such as indigocarmine, a water-soluble, blue indigoid acid dye that is used to visualize possible lesions of the urethra during surgical procedures. If the nucleic acids RNA and DNA are present in a drug, for example in the form of small interfering RNA, it is classified as an RNA/DNA therapeutic agent (22). Active agents based on modified T-cells or virus particles, for example, are classified as gene/cell therapeutic agents (21).

**Fig. 9:**
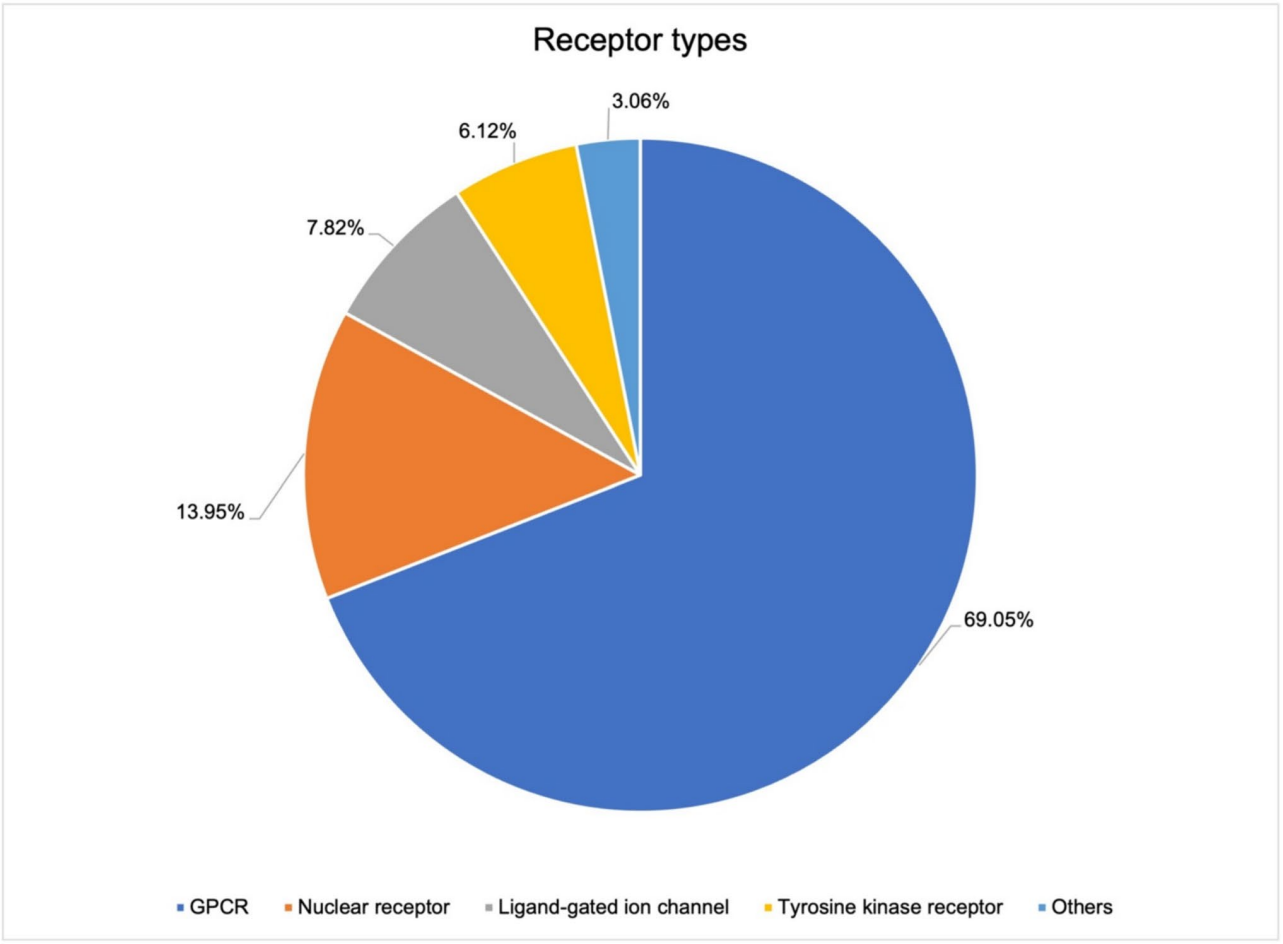
Specification of the receptor types in a total analysis of the 294 drugs acting on receptors. Blue shows the drugs acting on GPCRs, orange drugs acting on nuclear receptors, grey the drugs acting on ligand-gated ion channels, yellow the drugs acting on tyrosine kinase receptors, and light blue the drugs acting on other receptors

Of the total of 1110 newly approved drugs in the 37 years under review, 294 drugs act on a receptor as a target structure. Of these 294 drugs, 145 drugs acted as receptor agonists, 120 as receptor antagonists and 20 as receptor modulators. 9 drugs act as dual agonists and antagonists. Among the receptors, GPCRs are by far the most frequently addressed receptor class with 69.05% (see Fig. 9). Nuclear receptors such as steroid receptors are the target structure in 13.95%. 7.82% of the drugs are ligands at ligand-gated ion channels. 6.12% of the drugs are ligands at tyrosine-kinase-linked receptors. The group “others” (3.06%) includes for example transmembrane receptors such as Toll-like receptors as well as intracellular receptors that are not nuclear receptors (e.g. Nucleotide-binding Oligomerization Domain receptors).

**Fig. 10:**
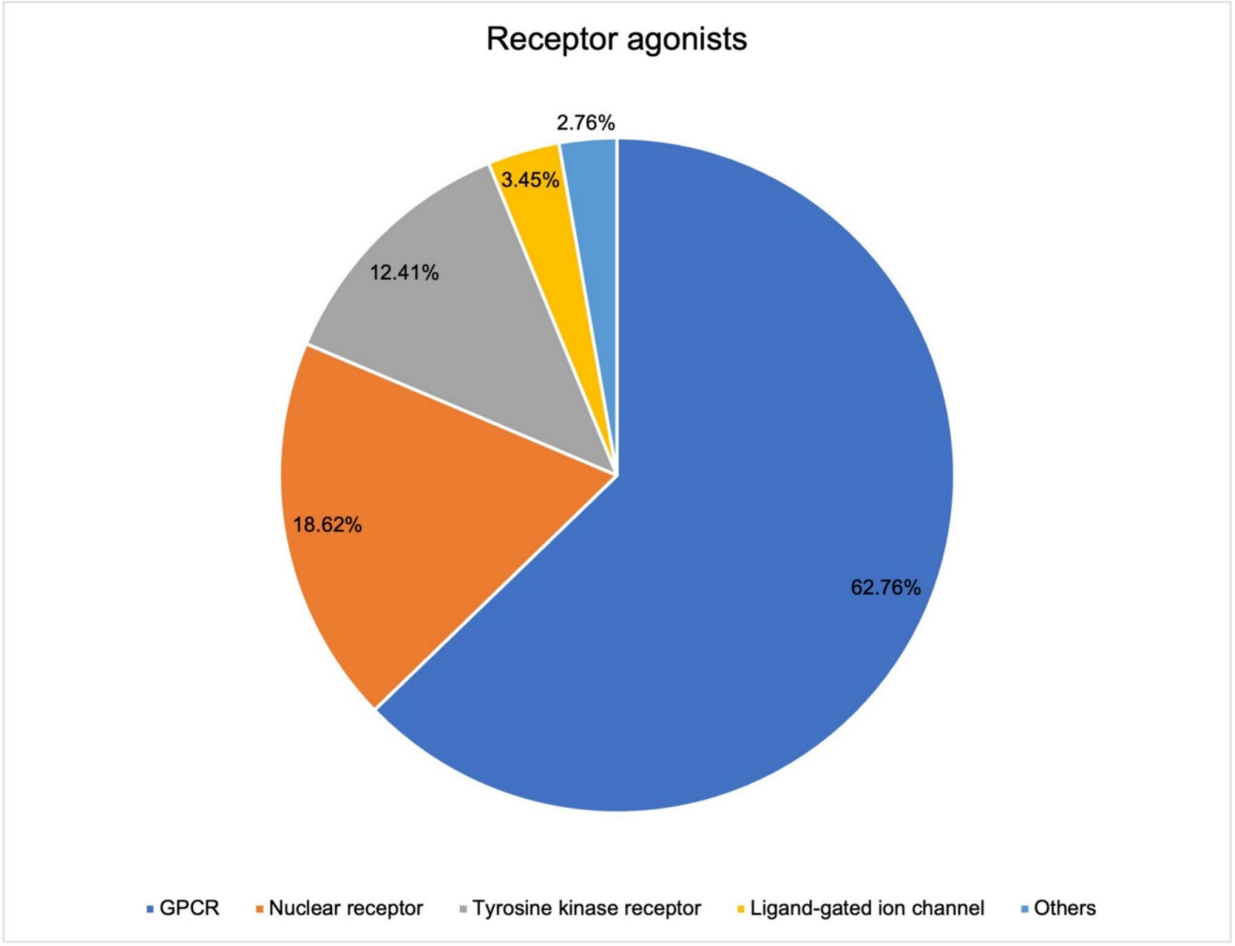
Differentiated receptor types among the receptor agonists. Subdivided into the five receptor types represented

Figure 10 shows the breakdown of receptor types among the agonists. The GPCRs are also by far the most common receptors among the agonists (62.76%). A higher proportion of agonists at nuclear receptors (18.62%) is noticed. There are several ligands at tyrosine kinase receptors (12,41%) but far fewer ligands at ligand-coupled ion channels (3.45%). Among agonists, only four drugs (2.76%) act on other receptors.

**Fig. 11:**
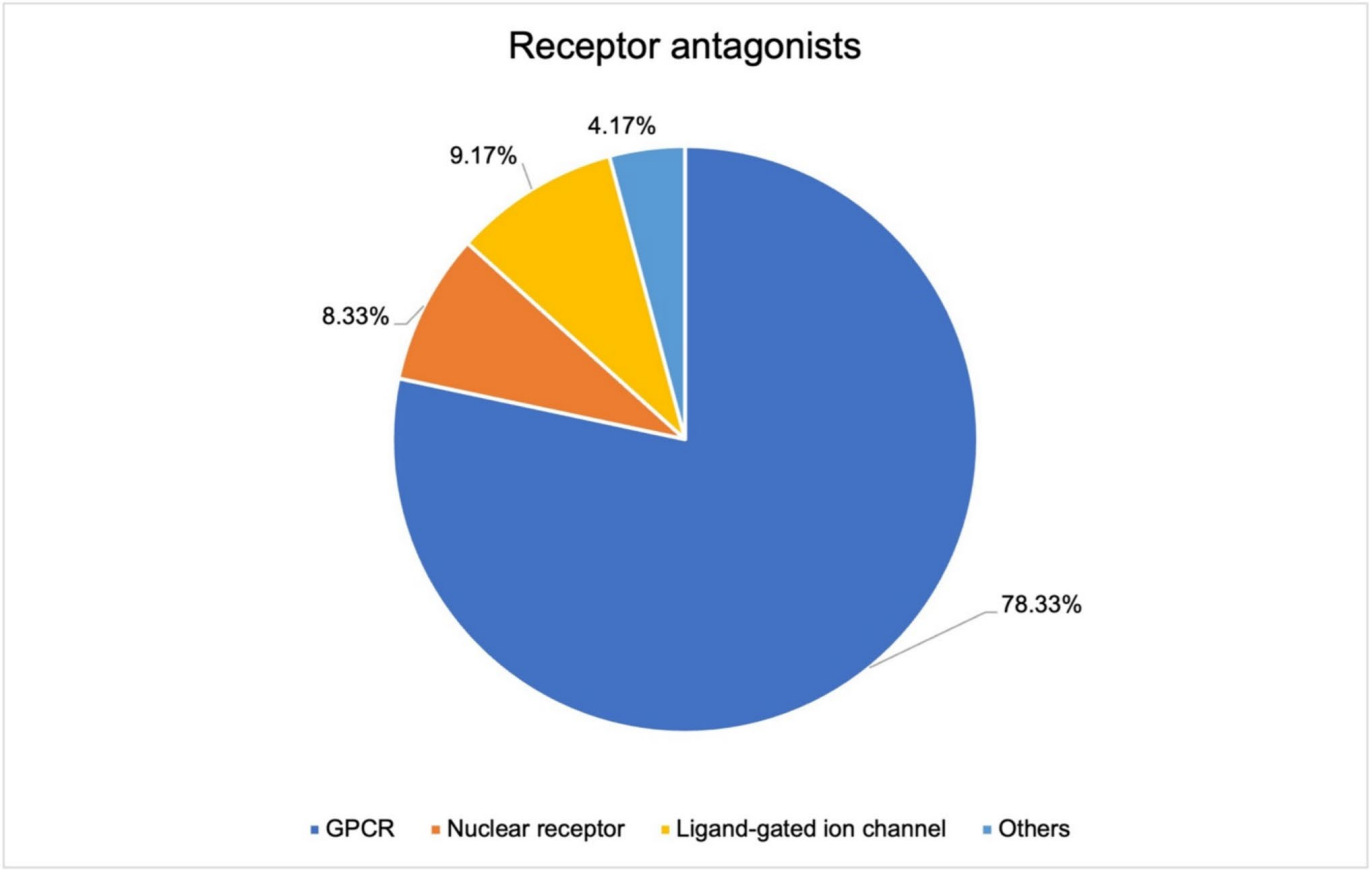
Differentiated receptor types among the receptor antagonists. Subdivided into the five receptor types represented

Figure 11 shows the proportions of the individual receptors among the antagonists. The GPCRs account for an even larger proportion (78.33%) of the receptors than in the analysis of total receptors. In contrast, nuclear receptors are underepresented. Drugs that act as antagonists on ligand-gated ion channels are represented to a similar extent. Other receptors are the addressed target structure of receptor antagonists in 4.17% of cases.

### Antibodies as a growing drug class

**Fig. 12:**
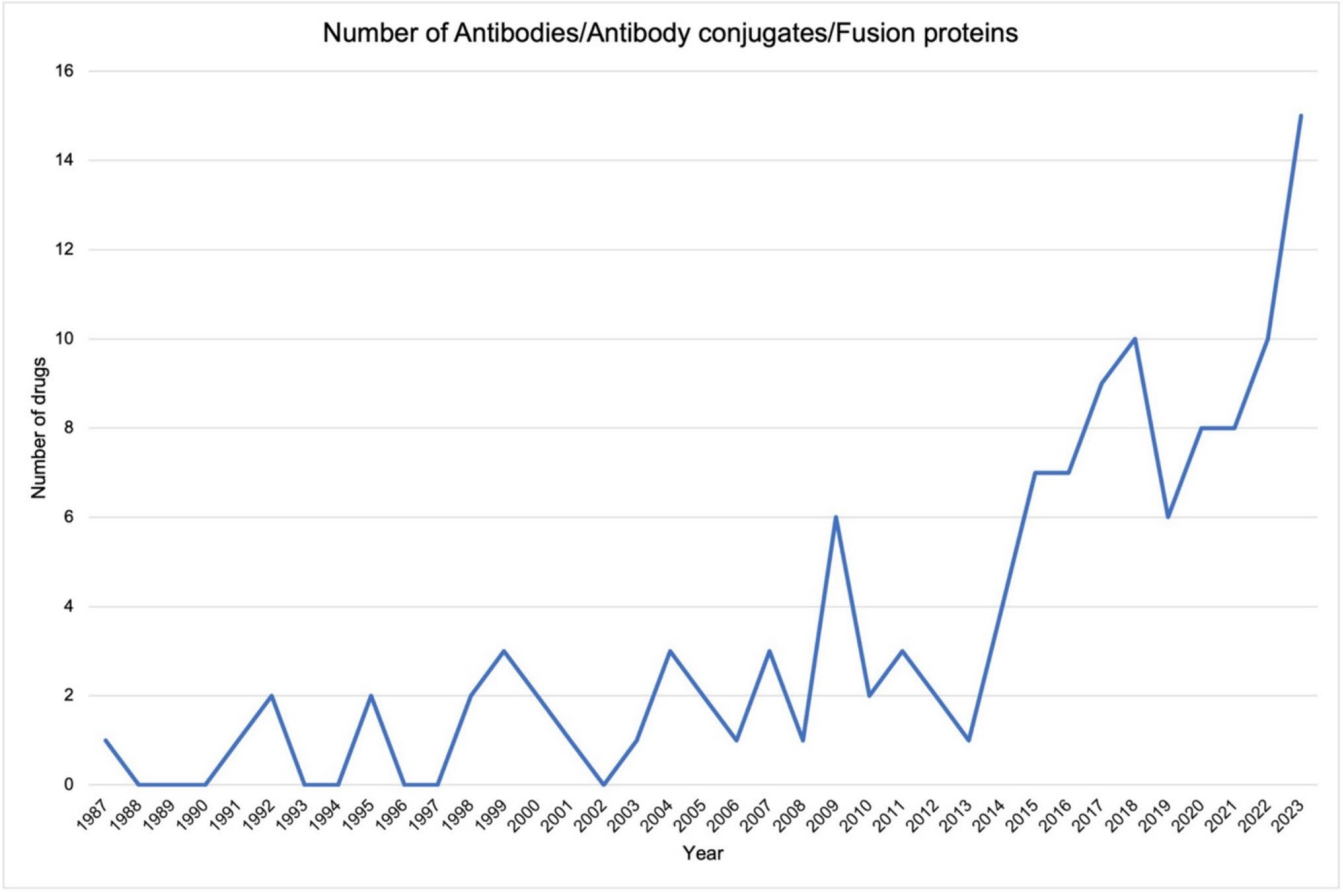
Number of approved antibodies/antibody conjugates and fusion proteins per year. Starting with the first year of analysis

Antibodies were the fourth most frequently addressed drug class in Germany from 1987–2023. Figure 12 shows that there has been an increase in approvals for this drug class, particularly since 2014. This figure lists antibodies, antibody-cytostatic combinations and fusion proteins. For a comprehensive list of drugs with the specific target from this group, see Table S1. Against this background, it is important to emphasize that antibodies can also act on GPCRs. These have now been marked with an asterisk (*) in Table S1. Antibodies that bind to a GPCR and thereby inhibit its function are not classic antagonists. For this reason, these drugs are listed in the antibody category but are nevertheless included in the total number of GPCR-drug approvals.

### Limitations and future studies

There are limitations in this study. Only drugs in the specified sources analyzed (Table 1 and Table S1) were studied. We considered only a drug under its international non-proprietary name (INN) and not multiple preparations containing any given drug except of nintedanib which was approved in 2015 under two different trade names and for different indications. One of these drugs has an oprhan status. Our study does not allow us to make a statement about all approved drugs worldwide. We included only representative databases. Our databases only represent the point of view at a specific point in time. None of the databases can provide a complete overview of all approved drugs in Germany. The Rote Liste only includes marketed drugs from companies that are organized in the Bundesverband (Federal Association) of the Pharmaceutical Industry in Germany, which can lead to a bias in the listing of drugs. The data on the percentage of GPCR drugs presented in Table 1 are certainly not comprehensive; they are meant to be representative for the overall perception. Tracing the origin of the 30% statement to the paper by Hopkins and Groom (2002), admittedly, has some speculative element, and it cannot be excluded that other sources for this statement exist. But this source is cited heavily, making it a probable source (Fig. 4). Our study has a retrospective character and cannot make forecasts for the future development of GPCR drugs.

Future studies should analyze additional data bases such as the drug lists of developed countries (e.g. Great Britain, the United States of America and Japan), emerging countries (such as Brazil and India) and developing countries (such as Sudan and Nigeria). Moreover, it will be very important to analyze the drug lists presented in standard textbooks from various countries. Based on the present study, however, it is not expected that a share of 30% of GPCR drugs will be reached in the next years. Considering drug approvals in recent years, it is anticipated that protein kinase inhibitors and therapeutic monoclonal antibodies (so-called “biologicals”) will continue to dominate new drug approvals. 

## Conclusions

GPCR drugs are often stated to contribute to 30% (and even more) of all approved drugs.

We identified a tentative literature source of the 30% value (Hopkins and Groom 2002) that was not substantiated by source data. Nonetheless, the 30% value was very attractive for GPCR researchers and adopted as gold standard. Based on this percentage, further analyses were carried out in the following years. These provide a much more precise definition of the databases analyzed. Nevertheless, the number of GPCR related drugs is often not sufficiently investigated regard to various databases and countries, particularly in the case of newly approved drugs.

By searching various databases, encompassing WHO drug lists, FDA lists, newly approved drugs over almost 40 years, national drug prescription lists and teaching lists we found the percentage of GPCR drugs in the 15–20% range much more realistic. Most newly approved drugs 1987–2023 are enzyme inhibitors. The number of approved antibodies has increased in recent years too. It is expected that this drug class will continue to grow substantially in the coming years.

This is a case study that shows how the frequent repetition of a statement in a large field of research leads to this statement being accepted as correct without checking the corresponding databases. We hope that our study encourages all researchers in their respective fields, beyond GPCRs, not to rely on “common knowledge” statements without further investigation. When stating percentages for GPCR-drugs, it is important to specify which database and country the source refers to. The focus of this study is explicitly on newly approved drugs in Germany. From 1987 to 2023, 19% of all newly approved drugs in Germany act on GPCRs.

## Supplementary Information

Below is the link to the electronic supplementary material.

Supplementary file1 (XLSX 272 KB)

## Data Availability

All source data for this work are available upon reasonable request.

## Abbreviations

AVR: Arzneiverordnungsreport (Drug Prescription Report)
EMA: European Medicines Agency
FDA: Food and Drug Administration
GPCR: G protein-coupled receptor
MHRA: Medicines and Healthcare products Regulatory Agency
NA: No data avaliable
IMPP: Institut für Medizinische und Pharmazeutische Prüfungsfragen (Institute for Medical and Pharmaceutical Exam Questions)
